# Impact of Label Noise on the Learning Based Models for a Binary Classification of Physiological Signal

**DOI:** 10.3390/s22197166

**Published:** 2022-09-21

**Authors:** Cheng Ding, Tania Pereira, Ran Xiao, Randall J. Lee, Xiao Hu

**Affiliations:** 1 Department of Biomedical Engineering, Georgia Institute of Technology, Emory University, Atlanta, GA 30332, USA; 2 INESC TEC-Institute for Systems and Computer Engineering, Technology and Science, 4200-465 Porto, Portugal; 3 Nell Hodgson Woodruff School of Nursing, Emory University, Atlanta, GA 30332, USA; 4 School of Medicine, University of California San Francisco, San Francisco, CA 94143, USA; 5 Department of Biomedical Informatics, School of Medicine, Emory University, Atlanta, GA 30332, USA; 6 Department of Computer Science, College of Arts and Sciences, Emory University, Atlanta, GA 30332, USA

**Keywords:** supervised learning, label noise, learning models, binary classification, Biomedical Signal

## Abstract

Label noise is omnipresent in the annotations process and has an impact on supervised learning algorithms. This work focuses on the impact of label noise on the performance of learning models by examining the effect of random and class-dependent label noise on a binary classification task: quality assessment for photoplethysmography (PPG). PPG signal is used to detect physiological changes and its quality can have a significant impact on the subsequent tasks, which makes PPG quality assessment a particularly good target for examining the impact of label noise in the field of biomedicine. Random and class-dependent label noise was introduced separately into the training set to emulate the errors associated with fatigue and bias in labeling data samples. We also tested different representations of the PPG, including features defined by domain experts, 1D raw signal and 2D image. Three different classifiers are tested on the noisy training data, including support vector machine (SVM), XGBoost, 1D Resnet and 2D Resnet, which handle three representations, respectively. The results showed that the two deep learning models were more robust than the two traditional machine learning models for both the random and class-dependent label noise. From the representation perspective, the 2D image shows better robustness compared to the 1D raw signal. The logits from three classifiers are also analyzed, the predicted probabilities intend to be more dispersed when more label noise is introduced. From this work, we investigated various factors related to label noise, including representations, label noise type, and data imbalance, which can be a good guidebook for designing more robust methods for label noise in future work.

## 1. Introduction

The success of supervised machine learning algorithms in classification tasks is dependent on a massive amount of training data, in spite of some alternatives that are being developing for learning models, such as flew-shot learning [1,2,3], or methods designed for learning with label noise [4]. Usually, in the medical field, human-annotated labels are [5] taken to be the ground truth. However, if there is noise in the labels, it undermines the robustness of any subsequent predictive models [6]. Label noise can arise in numerous ways. According to the work [4], the source of label noise can be categorized into four aspects: a. insufficient information; b. non-expert labeling; c. subjective labeling; d. data encoding problems. In our study, the label noise mainly comes from subjective labeling. In subjective labeling, label noise can be further divided into two categories: random label noise and class-dependent label noise. Random label noise could be introduced when annotation fatigue takes place leading to occasional random assignment of class labels. On the other hand, class-dependent label noise could be introduced when annotators have an unconscious bias or misunderstanding of annotation protocol for each class definition. The potential sources of label noise are even more complex in the case of medical data annotation. The rules for defining labels can vary enormously and may require physiological and clinical knowledge [6]. Label noise can get worse when more annotators are involved in the project because of the variabilities in the annotation ‘quality’ achieved by different annotators [7]. Meanwhile, the process is susceptible to high levels of inter- and intra-observer variabilities. One strategy to minimize the error in annotations is to have the same sample annotated by multiple annotators and then combine them to generate the final ground truth. Such an approach can mitigate the effect of subjective biases. However, the strategy is expensive and time-consuming, which in turn is rarely adopted for building large-scale training datasets and typically reserved for just the test set instead [4].

The major issue of label noise lies in the implication to classification performance of supervised learning algorithms. Several studies have investigated its potential impact and have shown that noisy labels can adversely affect the classification accuracy of trained classifiers [8]. However, some deep learning models appear to be robust against even high levels of label noise [9]. In view of the possible impact of label noise on the final classification, a number of novel algorithms have been developed that can tolerate certain amounts of label noise [6]. These include semi-supervised strategies, where all of the training data are used as data samples, but only the labels of selected samples are leveraged and the rest of the samples are treated as unlabeled [10]. In the work [11], *gray-zone* was defined when intracranial pressure (ICP) value range between normal and great risk, samples in *gray-zone* were considered as unlabeled, and then a semi-supervised approach was utilized. A similar framework was also conducted for the false alarm detection in patient monitoring system [12], which indicated that adopting semi-supervised learning with an unlabeled sample can be beneficial to both false alarm reduction and true alarm recognition rate. Another approach is to identify and filter the label noise to improve the quality of the training data [13]. A major drawback with removing training samples with noisy labels is that the removed samples may also be informative. In the medical domain, label noise in training data may propagate through the trained model to downstream clinical decisions, leading to medical errors that are difficult to find root causes for. Despite the significant implications of label noise, scant knowledge is available about the impact of label noise on biomedical data classification, with only a couple of studies dedicated to this topic [14,15]. In one study, a genetic algorithm-based method for mitigating label noise was developed that aimed to detect mislabeled samples during ECG signal classification [14]. Another study has suggested utilizing cross-validation to generate training data for five different classifiers. If the classification result of one sample from these five classifiers does not match its original label, they call it one mislabeled sample [15]. Both studies are targeted to identify and reduce label noise before deriving the final prediction model. To our knowledge, no studies have systematically investigated the impact of label noise on downstream classification performance from various machine models in the area of biomedical data classification.

In this study, both random and class-dependent label noises were artificially introduced into the training dataset to emulate the errors associated with human fatigue and annotation bias, which are the main sources of label noise in medical data annotation. The focus of this paper was to assess the robustness of different types of AI-based models to the label noise and bring the discussion of this relevant issue in the automatic approaches to the medical domain, which are usually dependent on the annotated data and the difficulties inherent in the labeling process. We investigate the impact of random and class-dependent label noise on the performance of machine learning (ML) and deep learning (DL) models, undertaking a binary classification task assessing the quality of photoplethysmography (PPG) signal. PPG is a popular tool to inform distal circulatory status and has gained its popularity recently years in cardiovascular research due to its ubiquitous presence in consumer wearable devices [16]. Its signal quality though can have a significant impact on downstream applications, which makes PPG quality assessment a great choice for examining the impact of label noise in the field of biomedicine. 

## 2. Materials and Methods

**Figure 1:**
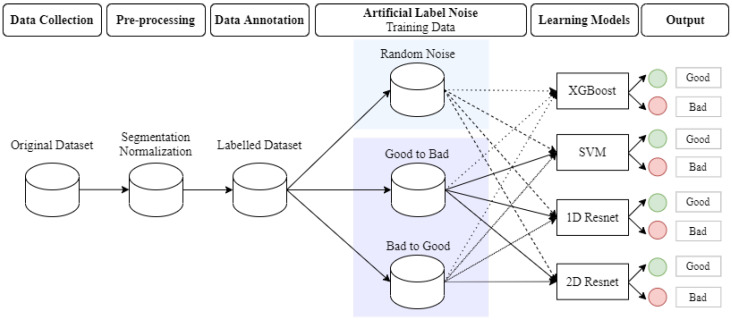
Overview of the pipeline of this study. Starting with data collection, followed by the annotation process of the quality assessment for each PPG segment and the experiments for the artificial label noise and the three learning models used in this study. The final output of the three learning models was analyzed and the results were compared.

This section describes the data collection and annotation process; the methods for artificial label noise introduced in the data, and the learning models used for PPG quality classification. The overview of this pipeline is presented in Figure 1. Figure 1 represents several parts of the pipeline developed to study the impact of the different label noise induced in the data. The PPG data were collected from several patients, and then segmented and normalized during the pre-processing staging; after that, each segment was labeled by the annotators. From the labeled dataset, part of the data has changed the label simulating the typical label noise, but in a controlled way with specific percentages for the labels changed. Three different learning models were used to classify the PPG segments, and the performance degradation was assessed following the increase of the label noise introduced on the data.

### 2.1. Training Data

The training data set consists of PPG recordings (sampled at 240 Hz) from 3764 adult patients admitted to the intensive care unit (ICU) at the University of California, San Francisco (UCSF) medical center between March 2013 and December 2016. Continuous PPG recordings were divided into non-overlapping 30-s records. We randomly selected 25 30-s PPG records from each patient, corresponding to a total of 78,278 30-s PPG records after preprocessing, as described in a previous study [17,18]. 

### 2.2. Test data

The test set consists of PPG recordings (sampled at 240 Hz) from 13 patients (age range 19 to 91 years, median = 73.5) admitted to the Neuro ICU at UCSF Medical Center between October 2016 and January 2018. The inclusion criteria of the selected patients consist of: (a) being diagnosed with acute ischemic stroke; (b) being at least 18 years old, and (c) English-speaking. Patients with significant problems relating to their attention, alertness, cognitive functions, or communication were excluded unless a legally authorized representative could give consent on their behalf. All the enrolled patients provided written consent after being informed of the protocols approved by the UCSF’s Institutional Review Board [18]. These patients were prospectively studied to test the validity of using a wrist band to collect PPG signals as compared to standard devices used at the bedside, but the data analyzed in this study were from the standard device that records PPG from fingers.

### 2.3. Annotation

The annotation process followed the same rules as defined in a previous study [17,18]. The 30-s PPG and synchronously recorded electrocardiogram (ECG) waveforms were presented to annotators to determine signal quality label (‘Good quality’, ‘Bad quality’ and ‘Not sure’) of each PPG record. Three strict conditions that qualify a PPG record as a good quality signal include: (i) its pulsatile waveform reflects blood volume changes in accordance with either physiological or pathophysiological (we expect ECG arrhythmia will cause irregular pulsatile PPG waveform) characteristics of the cardiovascular system; (ii) the waveform presents a consistent number of inflection points; and (iii) the waveform is free of irregularities that could not be explained by changes in the synchronous ECG. Records do not meet any of these three conditions will be considered as ‘Bad quality’. The records labeled as ‘Not sure’ were discarded from the following analysis. In the trial step, we assigned 100 random 30-s PPG signals to all the five annotators. Cohen’s kappa was utilized to assess the inter-rater agreement, the results showed a kappa coefficient of 0.87. Remaining PPG records were then assigned to each annotator without overlap for further annotation.

### 2.4. Artifact Proportion

PPG records in the test set were also annotated in terms of artifact proportion. This artifact proportion was defined by selecting all segments in a PPG record that were considered corrupted, then dividing their combined length by the total length (30 s) of the signal. The final distributions of labels in both training and test sets are shown in Table 1. The training set contained 30% bad quality records, with the test set presenting a very similar distribution.

### 2.5. Models

To investigate the impact of label noise on model performance, we adopted four different prediction models that take various representations of PPG signals as input. The learning models used in this work were selected from the previous works that already made an extensive comparison from different approaches [17,18]. The continuous PPG signal was first segmented into 30-s segments and normalized between zero and one before being transformed into three signal representations: 1D raw signal, 2D signal plot, and expert-engineered features [17]. To generate 2D signal plots, each PPG record was transformed into a 224 × 224 × 3 RGB image with a pixel density of 96 dots per inch (DPI). Residual networks have demonstrated robustness to label noise for various image classification tasks (ImageNet, MNIST, CIFAR-100) [19]. Therefore, the Resnet-18 was adopted as the model structure for samples represented as 2D signal plots. The 1D Resnet-34 was selected as the classifier for samples represented as 1D raw signal. Finally, two conventional machine learning algorithms, support vector machine and XGBoost were used for expert-engineered features (both temporal-domain and spectral-domain). Details about these features can be found in our previous study [17].

### 2.6. Hyperparameter Selection and Training Procedure

In our study, we use the same hyperparameter as our previous study [17,18]. For SVM, we use the RBF kernel. The optimal values for parameters C and sigma are 314.24 and 25.45. After tuning, we choose 4 as the max-depth for XGBoost and 10 as the number-of-round during training. For two deep learning models, we use the glorot_uniform as the initiation function for each convolution kernel. During training, the cross-entropy is used as the loss function and Adam as the optimizer with the learning rate 1 × 10^−4^. Each deep model is trained for 50 epochs, the final network parameter is chosen from the epoch with the least loss on the validation set.

### 2.7. Artificial Label Noise

To simulate complex situations that can arise during the human labeling process, two common types of label noise were artificially introduced in the training data in this study, so that the impact of label noise on the classification performance can be evaluated. One of the principal sources of label noise is fatigue or environmental distractions during the labeling process. The fatigue and environmental distractions can be considered random since they would not affect more one class than another, and their occurrence is not dependent on the data but on the characteristics of the annotator or/and the conditions under which the annotator is working. This kind of noise was simulated by using random label noise, with different percentages of randomly selected labels flipped to their counterparts (namely random flipping, i.e., for a selected sample, if the true quality label is ‘good’, it is flipped into ‘bad’ as label noise, and vice versa). To evaluate the impact of various levels of label noise, 10%, 20%, 30% and 50% of the labels from the training dataset were flipped to generate different versions of the training set.

Another common type of label noise is class-dependent noise which arises from intrinsic bias of annotators [20]. Here, the annotator tends to label one specific class incorrectly. To replicate noise of this nature, we selected a percentage of samples from only one class and flipped the labels. This procedure was repeated for the other class to see if the impact on the performance was different depending on the class of samples with biases. Again, 10%, 20%, 30%, and 50% of the labels were flipped for each class (i.e., good-to-bad flipping, and bad-to-good flipping) to produce different levels of label noise.

### 2.8. Experimental Design

Three types of experiments are designed to evaluate the various impact of label noise on the model performance. The objective of the first experiment is to compare the performance deterioration on three models caused by label noise. Four classifiers (SVM, XGBoost, 1D Resnet and 2D Resnet) are trained from training sets with three types of label noise (random flipping, good-to-bad flipping, and bad-to-good flipping) and four levels (10%, 20%, 30%, and 50%) of label noise and tested on the independent test set (see Figure 1). With each combination, the performance of the trained prediction model is generated. We use accuracy as the main performance metric in this study. Other metrics (such as sensitivity, specificity, positive predictive value, and negative predictive value) are also provided in the Supplemental Materials. In the second experiment, the objective is to compare the impact between three types of label noise on one single model. The same training process as the first experiment is implemented but only on one model. The third experiment is designed to investigate the influence of label noise on the model output probability. Besides predicting a binary label, the model output probability represents the model’s confidence on the prediction. With the availability of annotated artifact proportion, the third experiment is conducted by presenting the relationship of the artifact proportion with different models’ output probability. A 5-fold cross validation was conducted in each experiment, the mean value and variance of accuracy were reported in the result section.

## 3. Results

This section presents the performance of three binary classifiers including both ML and DL models trained on training data with three types of label noise and four levels of label noise.

### 3.1. ML vs. DL

**Figure 2:**
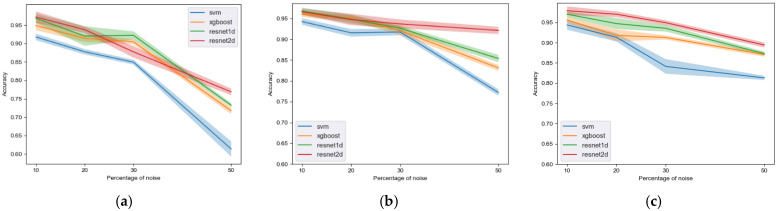
Performance of three classifiers according to levels of label noise in the training data. (**a**) Random flipping. (**b**) Good-to-bad flipping. (**c**) Bad-to-good flipping.

The prediction accuracy of all classifiers is presented in Figure 2 and summarized in Table 2. The deep learning models (green and red lines) proved to be more robust against all three types of noise than two traditional machine learning algorithms, including SVM (blue lines) and XGBoost (orange line) when it came to classifying good and bad quality PPG signals. In general, the performance gap was widened with increasing percentage of noise, indicating a greater decline in the performance of the SVM. When comparing the three types of label noise, random noise produced the greatest inter-model performance difference, with SVM suffering the most severe reduction in performance, and XGBoost suffering less but still worse than the two DL models.

Figure 2 demonstrates that DL models are affected by label noise to a lesser extent compared to the ML model, with the 2D Resnet as the most robust method of all three against label noise with superior performance over the other two methods in most of the simulated label noise. On the other hand, the conventional ML model SVM presents the greatest reduction in accuracy with label noise increasing from 10% to 50% (as large as 30.41% as shown in Table 2) as compared to DL models, indicating greater tolerance of DL models in handling label noise. Between two conventional models, XGBoost has slightly better tolerance than SVM with a 23% reduction in accuracy, other performance metrics, such as sensitivity, positive predictive value (PPV), and negative predictive value (NPV), generally present consistent patterns and are included in the Supplementary Materials.

### 3.2. Impact of Different Types of Label Noise

In the first experiment, the impact of each type of label noise was investigated based on their percentage in the training data. However, because the distribution of each class in the training data is different, the absolute number of noisy samples from each class could vary a lot. In this second experiment, three types of noise were considered and their impact on the classification performance was evaluated by examining the performance curves against the absolute number of flipped samples. This can be derived by converting the different percentage levels of label noise into the absolute number of samples per class to be flipped. The 1D Resnet model was selected to compare the performance impact of various label noise types, as it was the top performing model trained on the non-label flipping dataset shown in our previous study [18].

**Figure 3:**
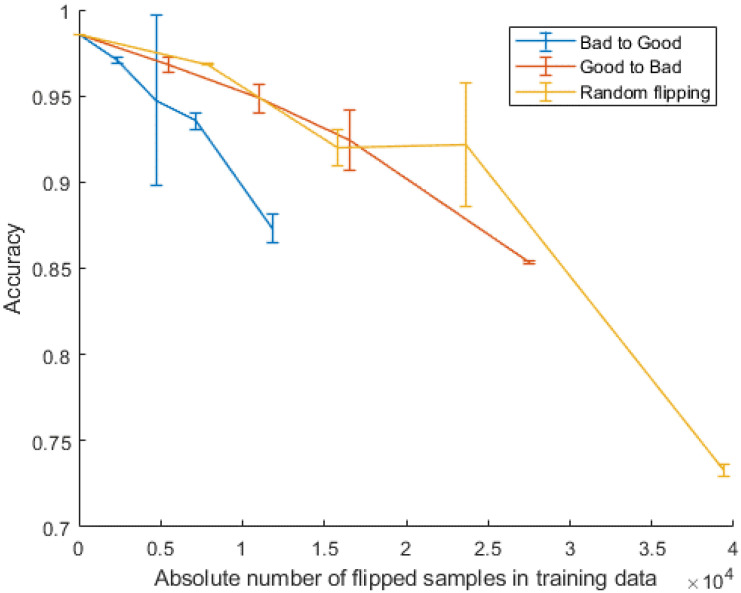
Impact of the absolute number of mislabeled records upon the best learning model on the cleanest training set from the previous work [17,18]. The three types of label noise were used and the performance degradation was verified, with different slopes accordingly to the type of noise induced.

Figure 3 presents the performance curves of 1D Resnet model trained across along varying numbers of flipped samples for each type of label noise. It shows the classifier had different levels of tolerance for different types of noise. When the absolute number of mislabeled samples was under 10,000, the trained 1D Resnet had greater robustness toward random flipping than annotator bias noise. For the two types of annotator bias noise, flipping from good-to-bad caused less confusion than flipping from bad-to-good. One plausible reason is that changing from bad-to-good corrupts the strict definition of good quality in this task. Introducing mislabeled poor-quality records will therefore have a major impact on the fundamental classification task.

### 3.3. Impact of Label Noise on Model Outputs

**Figure 4:**
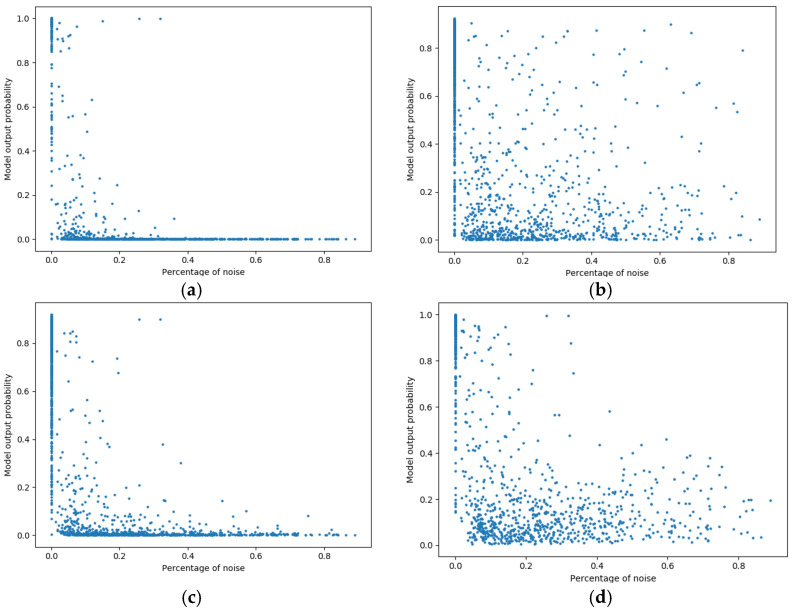
Scatter plots for the results of different probability predictions after adding a percentage of label noise to the PPG records in the initial training data: (**a**) trained on clean data; (**b**) trained with 10% random label noise; (**c**) trained with 10% good-to-bad switching as label noise; (**d**) trained with 10% bad-to-good switching as label noise.

To study the effect of label noise on classifier probability decisions, the annotation of artifact proportion for each PPG record in the test set was utilized [18]. We plotted the relationship between the classifier output probability and artifact proportion for each record in the test set. Here, larger model output probability indicates greater confidence of samples being classified as good quality. The 1D Resnet was used in this experiment. Four different classifiers were trained: the 1D Resnet trained on the original clean training data; and the same model structure trained on noisy training data with 10% of each of the three types of label noise (see Figure 4).

The model trained on the clean training data classifies PPG records with any nonzero artifact proportion as bad quality (i.e., an output with close to zero probability) because of the stringent definition of good-quality labeling (see Figure 4a). In comparison, when even a relatively small amount of label noise (i.e., 10%) was added to the training data, with either random or class-dependent label noise (see Figure 4b–d), the pattern of classifier prediction probability was changed. It became much more uncertain in general, with records with zero percent artifact showing a large range of probability outputs. This effect was found for all types of label noise.

In all cases where label noise was introduced, the distribution of the model output probability started to be more diverse than the distribution in Figure 4a. The addition of random label noise to the training set created a much more dispersed scatter plot indicating the output uncertainty of the classifier caused by the added randomness in training samples (see Figure 4b).

Within two class-dependent label noises, this effect was especially apparent when the label noise related to flipping the labels from bad-to-good. The plausible reason is that the classifier learned from mislabeled ‘good quality’ records which had a large range of percentage of artifacts, leading to the increased prediction probability on certain records with artifacts (see Figure 4d). In the case of the labels being flipped from good-to-bad, a diverse output probability was also presented but to a less extend comparing to the bad-to-good scenario.

### 3.4. Statistical Analysis

The pairwise Student’s *t*-tests are performed to compare the accuracies obtained in section A from different models. As reported in Table 3, the performance of these models is significantly different from one another after Bonferroni correction for multiple comparisons (*p* < 0.05).

To assess if there is a statistical difference between different types of label noise, pairwise *t*-test is utilized to compare the accuracy based on the 1D Resnet from three different label noise. As illustrated in Table 4, the impact of random noise is significantly different from class-dependent noise (*p* < 0.05). At the same time, the difference between two class-dependent noise types is not considered statistically significant (*p* > 0.05).

## 4. Discussion

Label noise is an inevitable issue in supervised learning tasks and the identification of source can come from many reasons. In this study, the annotation process was undertaken by domain experts, and the potential reasons for artifactual labels can be identified and summarized into random label noise and class-dependent label noise. The random label noise simulates the error caused when fatigue took place, leading to occasional random assignment of class labels, and the class-dependent label noise simulates the labeling errors generated from annotator bias raised from the misinterpretation of annotation rules. In this study, a binary classification framework was adopted to assess and classify signal quality of 30-s PPG records. Three types of label noise were simulated by controlling the type and amount of artificially flipped labels in the training sets, which were used to train different ML and DL classifiers. Such an experimental setup permits evaluation of the different tolerance of prediction models against label noise, different impacts on model performance from different types of label noise, and the subsequent effect on the confidence of model-predicted labels.

Between two conventional ML algorithms, the performance of XGBoost is less degraded by all three types of label noise than SVM, although they share the same input–hand-crafted features from our previous study [14]. The core of SVM is to use a Kernel to project the non-linearly separatable features into a high-dimension space where the features can be separated, and we do observe very close performance when the labels are all correct. However, when introducing noisy data samples, the label of support vectors is highly likely to be polluted and therefore the decision boundary is skewed. Compared to SVM, XGBoost shows better robustness because of its ensemble mechanism. Detailed mathematical derivation from [21] has demonstrated the relation between boosting and SVM, and it reports that in high-dimension space, the margins between XGBoost and SVM are drastically different. XGBoost intends to have a much larger margin so L1 norm in the loss function can ignore the irrelevant features and is therefore more robust to the label noise.

Both deep and traditional machine learning algorithms suffer from label noise. However, deep neural networks proved to be more accurate and robust against all three types of label noise we studied. As shown in Figure 2, when there was no label noise in the training data, the two deep classifiers performed slightly better than the SVM. When the level of label noise was increased, the deep classifiers not only maintained their superiority but also became proportionally better than SVM. The results confirm a broader hypothesis that deep learning models perform better than traditional machine learning algorithms in terms of their predictive power and robustness against label noise in the training data. It also suggests that deep models show greater promise for future works when a large dataset is available and label noise is a clear concern in the training data. There are two potential reasons: the first reason is proposed in [9], gradient updates can be easily canceled out for noisy samples while gradients from clean samples are more likely to contribute to the learning process. Another reason is the early stop mechanism, which will terminate the training process if the loss value on the validation set did not reduce for six epochs consecutively. This mechanism can drastically avoid the overfitting and thus performance degradation.

Between the two deep learning models, the 2D model was more robust than the 1D model. When we increased the noise level from 20% to 30% in the bad-to-good type of noise, the 1D model showed an unexpected improvement in terms of accuracy. Nonetheless, the 2D model was still slightly superior to the 1D model. One plausible reason could be that the 1D model had 34 layers, which is deeper than the 18 layers in the 2D model. In the machine learning bias-variance tradeoff theory, a deeper model can more easily adapt to training data with more variance, leading to a reduction in generalizability and an increment of error in the test data. The better performance of the 1D model when there was no label noise further supports this hypothesis.

A significant difference in performance was also found within the three different types of noise when the same classifier was adopted. As shown in Figure 3, when the absolute number of noisy samples was 10,000 in the training data, the bad-to-good flipping resulted in about 9% more reduction of accuracy than the other two types of noise. One plausible explanation is that there was an imbalance in samples from two class conditions in the original training data, with only 30% of the samples being labeled as bad quality. Bad-to-good flipping scheme exacerbates the sample imbalance issue, leading to a reduction in classifier performance. Additionally, changing from labels from bad-to-good quality breaks the restricted definition of good quality signals. Specifically, introducing poor quality signals into good-quality condition sabotages the classifier’s ability to learn discriminative features truly separating the two quality conditions, which in turn leads to a dramatic decrease in classification performance.

The calibration ability of the classifier was also vulnerable to label noise, which has been even less studied. We found that even 10% of each type of noise could significantly change the classifier output probability (see Figure 4). Due to the restricted rules imposed for good quality signals in our study, a 30-s PPG record with even a small percentage of artifacts would still be labeled as bad quality. This restriction is reflected in Figure 4a, where most samples with other than zero percentage of noise are assigned with a small probability of being good quality signals. However, when 10% label noise was introduced into the training data (Figure 4b–d), many samples with nonzero percentage of noise were assigned with a higher probability of being good quality signals, resulting in a more spread pattern of the noise-probability map. The results suggest label noise alters model probability calibrations and that a more sophisticated strategy is required for recalibrating the classifier output.

### Limitations

The current study represents an evaluation of the impact of label noise on the accuracy and calibration of binary classifiers with a case study of PPG signal quality. However, restrictive definitions for labeling physiological events are highly common in the medical domain. In this study, the label definition of ‘good quality’ class is also strict, which makes the distribution of model output probability skewed to the bottom left instead of evenly distributed. The conclusions of this work may need to be limited to contexts where the class labels are based on a very specific definition in the classification problem. As the study was limited to binary classification, a similar study focused on multiclass classification would also expand the range of possible findings. The current study was focused on the several types of label noise that could be found in the medical data; however, the combination of them was not studied at this time, since the main objective was to compare the robustness of different types of learning models to each individual label noise. The impact of the combination of the label noise on the learning models’ performance will be assessed in future work.

This study focused on using the quality assessment of individual 30-s PPG records as a problem to study label noise. This enabled us to explore the influence of different types of noise potentially arising from the human annotation process. The generalizability of our conclusions would be reinforced by examining a wider range of PPG-based tasks. It is also the case that different tasks may be susceptible to different types of noise in the data set. This suggests a need for a more task-specific investigation.

The current work was dedicated to assessing the robustness of the previous developed classification models; however, other recent promising approaches have been proposed to deal with sequential time series such as transformers. Transformers and other AI-based models must be continually explored trying to find the best classifier for the quality assessment. Also, our results show deep learning methods are more robust to label noise, our future work would also focus on further improving robustness of deep neural networks.

## 5. Conclusions

Label noise is a daunting challenge for supervised learning. In this situation, there is a greater risk of classifiers learning from mislabeled records. An ideal classifier should be able to distinguish between patterns of representative data and label noise. Deep learning has shown to be better at handling a certain amount of label noise than shallow machine learning models, of which Resnet is particularly robust against noisy labels. This work has compared the influence of label noise on a classification task aiming to assess the quality of physiological signals using different learning algorithms. The results have shown that deep learning-based models can deal with larger amounts of label noise than traditional machine learning algorithms, with less degradation in their performance. With regard to different types of label noise, learning models are more heavily affected by annotation bias than random noise, in particular towards a minority class. This underscores the importance of selecting more robust models that can deal with real-world label noise. To sum up, the present study systematically characterizes the impact of various types of label noise on model performance and probability calibration and provides valuable insight into model selection that could mitigate the issue of label noise.

## Figures and Tables

**Table 1:** The distribution of signal quality in the analyzed patient cohorts.

Data	Number of Patients	Number of Records	Bad Quality Records	Good Quality Records
ICU—Training set	3764	78,872	23,764 (30%)	55,108 (70%)
Neuro ICU—Test set	13	2683	815 (30%)	1868 (70%)

**Table 2:** Mean accuracy values in relation to an increase in label noise in the training data.

Accuracy (Random Flipping)				
**Percentage of noise**	**1D Resnet**	**2D Resnet**	**SVM**	**XGBoost**
10%	96.84% ± 0.01	97.20% ± 0.008	91.78% ± 0.01	94.88% ± 0.01
50%	73.26% ± 0.02	76.69% ± 0.09	61.37% ± 0.18	71.75% ± 0.01
**Accuracy (Good-to-Bad)**				
**Percentage of noise**	**1D Resnet**	**2D Resnet**	**SVM**	**XGBoost**
10%	96.79% ± 0.01	96.61% ± 0.007	94.26% ± 0.01	95.99% ± 0.01
50%	85.38% ± 0.2	92.13% ± 0.01	77.20% ± 0.13	83.10% ± 0.01
**Accuracy (Bad-to-Good)**				
**Percentage of noise**	**1D Resnet**	**2D Resnet**	**SVM**	**XGBoost**
10%	97.06% ± 0.01	97.89% ± 0.003	94.38% ± 0.01	95.60% ± 0.01
50%	87.30% ± 0.13	89.42% ± 0.05	81.30% ± 0.03	87.07% ± 0.01

**Table 3:** Statistical result between different models based on pairwise *t*-test.

Model	1D Resnet		2D Resnet		SVM		XGBoost	
	t-Statistic	*p*-Value	t-Statistic	*p*-Value	t-Statistic	*p*-Value	t-Statistic	*p*-Value
1D Resnet	-	-	2.5282	0.0142	8.6594	<0.05	12.6235	<0.05
2D Resnet			-	-	9.8804	<0.05	11.9236	<0.05
SVM					-	-	7.2637	<0.05
Xgboost							-	-

**Table 4:** Statistical result between different label noise on pairwise *t*-test.

Label Noise	‘Bad to Good’		‘Good to Bad’		‘Random’	
	t-Statistic	*p*-Value	t-Statistic	*p*-Value	t-Statistic	*p*-Value
‘bad to good’	-	-	0.9127	0.2625	3.2615	<0.05
‘good to bad’			-	-	3.3762	<0.05
‘Random’					-	-

## Data Availability

The data that support the findings of this study are available from UCSF but restrictions apply to the availability of these data, which are now made available to investigators at Emory University under a data use agreement between the two institutions, and so are not publicly available. Data may be available from the authors upon reasonable request and with institutional permission of UCSF. The codes that support the findings of this study are available from the corresponding author upon reasonable request.

## Supplementary Materials

The following supporting information can be downloaded at: https://www.mdpi.com/article/10.3390/s22197166/s1, Table S1: The comprehensive performance of different models with different type of label noise.
